# Review: spinal shock, crossed extensor reflex, and mass reflex in dogs and cats—a literature review, clinical observations and considerations

**DOI:** 10.3389/fvets.2026.1703941

**Published:** 2026-02-09

**Authors:** Koen M. Santifort, Dakir Polidoro, Michelle Hermans, Agnieszka Olszewska, Adriana Kaczmarska, Federica Poli, Jos Bongers

**Affiliations:** 1 IVC Evidensia Small Animal Referral Hospital Arnhem, Neurology, Arnhem, Netherlands; 2 IVC Evidensia Small Animal Referral Hospital Hart van Brabant, Neurology, Waalwijk, Netherlands; 3 AniCura Veterinary Practice Plantijn, Neurology, Antwerp, Belgium; 4 AniCura Vetimacs Medical Imaging, Brussels, Belgium; 5 Nesto Veterinary Referral Center Orion, Neurology, Herentals, Belgium; 6 Fachtierarztzentrum Cologne Merheim, Cologne, Germany; 7 School of Biodiversity, One Health and Veterinary Medicine, University of Glasgow Small Animal Hospital, Glasgow, United Kingdom; 8 Valdinievole Veterinary Clinic, Pistoia, Italy; 9 IVC Evidensia Moorview Referrals, Cramlington, United Kingdom

**Keywords:** cutaneous trunci reflex, hyperreflexia, myelopathy, patellar reflex, spasticity, withdrawal reflex

## Abstract

The phenomena of spinal shock, crossed extensor reflex, and mass reflex are discussed to different extents in veterinary neurology textbooks, in literature on experimental animal models, and clinical reports on veterinary neurological patients. Aspects related to timing of onset of spinal pathology, their implications for neuroanatomical localizations, and their prognostic significance are clinically relevant. In this review article, the authors discuss and summarize the current literature and highlight collective observations in clinical veterinary neurology patients. Underreported observations emphasized in this article include the possibility of (1) asymmetrical/lateralized/unilateral spinal shock, (2) crossed extensor and mass reflexes in patients with (per-) acute myelopathies, (3) asymmetrical/lateralized/unilateral crossed extensor reflex, (4) mass reflexes observed in patients with (per-)acute myelopathies and concurrently with signs of spinal shock, and (5) a pelvic limb crossed extensor reflex in patients with pathology other than cranial to the lumbar intumescence.

## 1 Introduction

The neurological examination of veterinary patients is paramount to determine (A) whether there is a neurological disorder, (B) what part of the nervous system is involved (neuroanatomical localization), (C) what the main differential diagnostic categories are, and (D) the severity of the disorder at hand (1). Segmental spinal reflex tests are a key component of the neurological examination. In textbooks, literature on experimental animal models, and literature on clinical veterinary neurological patients, the phenomena of spinal shock, crossed extensor reflex, and mass reflex are discussed to a variable extent as will be detailed in this review. Aspects related to timing of onset of spinal pathology, their implications for neuroanatomical localizations, and their prognostic significance are clinically relevant. In this review article, the authors discuss and summarize the current literature and highlight collective observations in clinical veterinary neurology patients. Underreported observations emphasized in this article include the possibility of (1) asymmetrical/lateralized/unilateral spinal shock, (2) crossed extensor and mass reflexes in patients with (per-)acute myelopathies, (3) asymmetrical/lateralized/unilateral crossed extensor reflex, (4) mass reflexes observed in patients with (per-)acute myelopathies and concurrently with signs of spinal shock, and (5) a pelvic limb crossed extensor reflex in patients with pathology other than cranial to the lumbar intumescence. Case examples are provided to illustrate these observations.

## 2 Pathophysiology and experimental data

### 2.1 Spinal shock

Spinal shock (SS) is defined as a transient loss of spinal reflex activity and muscle tone below the level of an acute spinal cord lesion due to interruption of descending facilitatory pathways (2); its characteristics evolve over time. They range from complete absence of both monosynaptic deep tendon reflexes (e.g. patellar reflex) and polysynaptic cutaneous (“superficial”) reflexes (e.g. perineal reflex) to variable combinations of retained, partially recovered and absent spinal reflexes (2). For example, the patellar reflex typically returns before the withdrawal reflex (2). Its severity correlates with the extent of spinal cord disruption, whether functional (physiological) or structural (anatomical), and is absent in slowly progressive injuries (2). SS is believed to result from synaptic alterations below the lesion, including enhanced presynaptic inhibition, a four-fold rise in inhibitory neurotransmitter glycine, and motor neuron hyperpolarization, creating a transiently misleading clinical picture of a lower motor neuron lesion (2–4).

The phenomenon arises from disrupted descending modulation of spinal interneurons and motor neurons via the corticospinal, rubrospinal, vestibulospinal, and reticulospinal tracts (5). Electrophysiological studies support the presence of supraspinal segmental inhibition during SS, showing blocked mono- and polysynaptic reflex arcs (6, 7). Loss of muscle tone and reflexes may also stem from dysfunction of the γ-efferent system, which controls muscle spindle sensitivity; loss of tonic descending input reduces γ-motor neuron activity, lowering spindle excitability and reflex gain (8). The more pronounced SS seen in species such as primates suggests evolutionary younger descending tracts are predominantly involved (9). In humans—where SS has been most extensively characterized—four clinically differentiable phases are described: initial areflexia (the phase mostly referred to as “spinal shock”), return of reflexes, early hyperreflexia, and spasticity (10). Spinal shock resolves over days to months, gradually transitioning to spasticity in many cases (2, 10, 11). If defined by the initial recovery of any reflex, it may last only 20 min to 1 h; however, if based on the absence of deep tendon reflexes, it can persist for several weeks (11).

Experimental studies have made use of rabbits, dogs, and cats as models for studies of SCI and SS (12–14). Most of these studies have focused on the aspect of timing in relation to spinal reflex return. This SS phase can be easily missed in veterinary medicine due to the comparatively fast recovery (compared to humans) and the fact that patients are often not presented to a veterinarian as quickly as humans after acute spinal cord trauma. In the experimental study of Blauch (12), the patellar reflex took between 30 min and 2 h to recover. The withdrawal reflex was found to take the longest to recover, ranging from 12 h to 6 weeks. Spinal shock and associated metabolic changes have been documented in macaque monkeys following T10 spinal cord transection; during the acute phase of spinal shock, glucose metabolism was increased in superficial dorsal horn regions and decreased in the ventral motor layers, while post-reflex recovery showed increased metabolism across all Rexed layers, especially layers I, V, and VI (15). Another report noted that two monkeys exhibited brief spinal shock (< 1 week), marked by flaccid paralysis and absence of lower limb reflexes (16). In a broader reflex review, it is noted that spinal shock is pronounced in primates due to their strong descending inhibitory control (2).

In these experimental studies, lateralization of SCI and its effect on SS are often not well recorded or described.

### 2.2 Crossed extensor reflex

The crossed extensor reflex, first systematically described by the British neurophysiologist professor Charles Scott Sherrington in early 1900s, remains fundamental in understanding spinal cord function and interlimb coordination (8). Sherrington demonstrated that stimulation of afferent nerves in one limb could elicit a reflex extension in the contralateral limb, even in the absence of conscious brain activity (9).

Stepping movements, including the rhythmic alternation between extension and flexion of the limbs, are primarily mediated by spinal reflex circuits and central pattern generators (17). The crossed extensor reflex plays a crucial role in this coordination, allowing contralateral limb extension during ipsilateral limb flexion to maintain balance during locomotion and standing position (16, 17). Under normal circumstances, this reflex is inhibited in recumbent animals, as weight support is not required (17, 18). However, in some animals with spinal cord lesions, this inhibition is lost, resulting in contralateral limb extension in response to limb flexion (17).

Physiologically, the crossed extensor reflex is a polysynaptic reflex, which means that interneurons are involved in addition to sensory and motor neurons (18, 19). These interneurons facilitate signal divergence by relaying afferent input to an extensive population of efferent neurons (18, 19). This mechanism allows a single input modality to concurrently elicit excitation of agonist muscles and inhibition of antagonists (18, 19). When descending motor pathways are affected by a lesion such as an IVDE or ischemic myelopathy, all, several or more rarely specific tracts may be involved. This results in disinhibition of the motor neurons of the intumescence(s). In short, the pathological expression of the crossed extensor reflex can be understood as disinhibition phenomenon caused by a spinal cord disorder (20). In this scenario, testing the withdrawal reflex in one limb is enough to excite motor neurons in the contralateral side of the spinal cord. If a dog exhibits a crossed extensor reflex in lateral recumbency, an upper motor neuron lesion or dysfunction of their descending spinal tracts should be suspected (18). Most of these patients will exhibit clinical signs suggestive of spinal cord pathology (18, 20). However, it is important to recognize that the crossed extensor reflex may involve two mechanisms: propriospinal and recurrent loop supraspinal pathways (21). Consequently, lesions involving brainstem upper motor neuron pathways could also contribute to the manifestation of this reflex (21). The experimental literature provides important context for understanding these mechanisms. Sherrington's work in spinal and decerebrate cats first characterized contralateral extensor activation during ipsilateral flexion and established the crossed extensor reflex as an integral component of spinal integrative function (22–26). Subsequent experimental studies confirmed that the reflex may be diminished or absent immediately after cord transection, likely reflecting acute spinal shock, but re-emerges as intrinsic spinal circuits adapt over time (27). Eccles demonstrated that crossed extensor responses in decerebrate, deafferented cats are shaped by interacting excitatory and inhibitory processes (28). Together, these experimental studies support the view that the crossed extensor reflex is fundamentally spinal in origin, with supraspinal pathways providing additional modulation when intact.

In large animals, the crossed extensor reflex is considered normal in newborn ruminants and horses whose upper motor neuron pathways are not yet fully developed and resolves spontaneously over the early postnatal weeks (29). However, in adults, the presence of a crossed extensor reflex is considered abnormal and suggests a disruption in normal upper motor neuron function (29, 30). The presence of crossed extensor reflex in large animals is anecdotally usually observed with severe and chronic central motor pathway lesions (29, 30). Such animals typically exhibit markedly reduced voluntary motor activity in the affected limbs that are being tested (29). In non-mammalian species, crossed extensor reflexes have also been documented; crossed extensor reflex is reported in the pelvic limbs of a flamingo (Phoenicopterus roseus) that was presented in ventral recumbency after a sudden onset of bilateral non-ambulatory pelvic limb paresis due to a T5–6 discospondylitis (31).

### 2.3 Mass reflex

The term “mass reflex”, historically attributed to Dr. George Riddoch, a pioneer in SCI research alongside Dr. Ludwig Guttmann, refers to a constellation of reflexive responses (sometimes referred to as the “Riddoch mass reflex”) observed below the level of a severe SCI (32–34). Though classically described in human medical literature as a chronic phenomenon (33–37), the term's definition and clinical implications remain inconsistently applied across both human and veterinary medical literature.

In the human literature, the mass reflex has been used mainly to refer to various reflex activity including visceral motor, somatic motor, and autonomic activity observed in humans predominantly in the regions of the body innervated by segments of the spinal cord caudal to a level of severe SCI (34–37). Some texts now refer only to autonomic dysreflexia when the term mass reflex is used (as a synonym) or do not use the term “mass reflex” at all (36, 37). Autonomic dysreflexia entails manifold expressions of overactivity, or rather dysregulation, of discharge of the autonomic nervous system (predominantly sympathetic effects are described, such as hypertension) (34–37). The reflex is associated with severe SCI, as the loss of most or all supraspinal input permits spinal circuits to function without regulation, leading to increased excitability (34–37). In addition, the reflex has been implied to be a chronic phenomenon as increased reflex excitability and loss of supraspinal inhibition have been described to occur following the recovery of spinal shock and the start of neuroplastic changes (37).

In veterinary medicine, documentation of mass reflexes remains sparse and there is currently no clear definition. We propose a working definition suitable for veterinary neurology: the mass reflex is characterized by involuntary autonomic and/or somatic motor activity in body regions innervated by spinal segments caudal to a severe SCI reflexively occurring after a (typically external) stimulus. An internal stimulus (i.e. the passage of feces through the rectum) might also elicit mass reflex activity. Mass reflex activity may include one or several of the following: sustained (tonic) or repeated (clonic) pelvic limb, tail, abdominal, and perineal muscle contractions, as well as visceral events such as urination and defecation. The variable extent of these may subjectively be interpreted as mild–severe. We recognize that this definition is imperfect, however, as it leaves room for discussion on what extent should be considered a lower limit for calling it a mass reflex. We distinguish this phenomenon from autonomic dysreflexia which is not well-documented in veterinary medicine. This is not to say that autonomic dysreflexia does not occur. However, it is currently underrecognized and not routinely monitored for, and therefore its presence in naturally occurring spinal pathology in veterinary species cannot be well-ascertained.

Mass reflex activity in clinical veterinary patients is primarily described in textbooks to be observed in cases of thoracolumbar (T3–L3) SCI, with clinical hallmarks including exaggerated or prolonged flexor responses in the pelvic limbs and tail, often initiated by noxious stimuli such as firm pressure or pinching (20, 38). The authors observations' also include tonic or rhythmic contraction of the tail (“wagging”), lumbar (repeated lumbar flexion and extension), and abdominal musculature (repeated tension of the abdominal muscles), as well as visceral signs such as involuntary urination and defecation due to reflex contraction of the bladder and rectum. Perineal and genital muscular contractions (e.g., bulbocavernosus, constrictor vulvae) may also be evident on close examination. The authors have also noticed considerable variability in presentation, with these responses fluctuating over time, thereby adding to diagnostic ambiguity.

The work of Dr. C. S. Sherrington and colleagues has been pivotal in the understanding of reflex development after brain injury or SCI (9, 14, 17, 26, 39–41). Various aspects regarding timing and nature of reflexes after experimental lesions to the spinal cord and brain are included in his publications. From his descriptions and those of others, it is clear that some features of those reflexes, in both early and late stages after injury, entailed what we include here in the term “mass reflex” (9, 14, 17, 26, 39–41). In that sense, a mass reflex is nothing more than a most prominent form of hyperreflexia. The timing of reflex development after experimental injuries in rodents, cats, and primates differs, and that of clinical veterinary and human patients without doubt does as well (26, 39–44).

Human studies corroborate that spinal reflex excitability generally increases over time post-injury (45–49). Although most research focuses on chronic stages, there is growing recognition that reflex evolution and spasticity (quantified in scales), rather than single reflex markers, offer superior prognostic value (2, 10, 33, 50–53). As a result, the development of spasticity, quantification thereof, and associated reflex patterns in humans has been linked to better functional motor outcomes, although spasticity can also be detrimental for daily life and a cause of pain (53–55). In clinical veterinary neurology, a spasticity scale for dogs has also been described, potentially offering a useful tool for outcome measurement or guiding of (physio)therapeutic measures (56).

## 3 Clinical veterinary literature

### 3.1 Spinal shock

SS is reported in 6%−66% of dogs with acute thoracolumbar spinal cord injury (SCI) (57–60). In a clinical setting, SS is so far only reported in dogs with acute SCI with the exception of one case with lesions in the brain stem and cervical spinal cord both, consisting of necrotizing fibrinoid thrombotic arteritis and secondary rarefaction, gliosis, necrosis and hemorrhages (57–66).

McBride et al. (65) and Gouveia et al. (59) found acute intervertebral disc extrusion (IVDE) to be the most prevalent etiology for SS in dogs. In contrast, Full et al. (57) and Hodshon and Thomas (58) found ischemic myelopathy [presumed fibrocartilaginous embolism (FCE)] and acute non-compressive nucleus pulposus extrusion (ANNPE) as the most prevalent etiologies.

SS is most often recognized in cases with a referral presentation within 24 h after injury, most often with decreased or absent withdrawal reflexes (76%−100% of cases) (57, 60, 65), patellar reflexes (24%−40%) (57, 60, 65), muscle tone (24%−53.3%) (60, 65), perineal reflex (26.7%) (60), anal tone (20%) (60), or a combination of the above.

Clinically, as a main goal of the neurological examination is to yield a correct neuroanatomical localization of the lesion(s), it is highly relevant to differentiate between an upper motor neuron lesion with SS or a lower motor neuron lesion. Findings that lead to prioritizing a T3–L3 myelopathy with SS over an L4–S3 include (1) the combination of an absent withdrawal reflex and normal patellar reflex (although the patellar reflex can be absent or weak as well), (2) a cutaneous trunci reflex cut-off, (3) the localization of hyperesthesia on palpation of the vertebral column and paraspinal musculature, and (4) the distribution of the loss of nociception (e.g. entire pelvic limb or distribution pattern related to segments included only in L4-S3) (57, 58, 65). For the cutaneous trunci reflex cut-off, McBride et al. found that 76.3% of cases had a cutaneous trunci cut-off in a T3–L3 myelopathy, and only 30.7% in an L4–S3 myelopathy (65). Dogs with a cutaneous trunci cut-off were 8.8 times more likely to have a T3–L3 myelopathy. They also found that paraplegic dogs were 7.87 times more likely to present with SS than paraparetic dogs, and dogs with decreased pelvic limb tone were 88% less likely to have SS, supporting a true L4–S3 lesion (65).

For observations regarding SS recovery, Full et al. (57) reported that 93% of dogs with SS had improved or normal reflexes by 60 days post-injury, with 88% already improved between 1–12 days. Hodshon and Thomas reported the patellar reflex to return to normal within a mean of 12 h and the withdrawal reflex had a mean recovery time of 66 h in patients with acute thoracolumbar lesions. Another study reported >48 h for the withdrawal reflex to recover (58).

To date, multiple studies found no association between SS and recovery of ambulation (57, 58, 60, 62, 65). A single study by Gouveia et al. (59) reported that 66.9% of dogs with SS recovered ambulation compared to 97.6% in controls. Mean time to regain ambulation was 31.57 days in the SS group vs. 23.02 days in controls.

While most of the literature discussed SS in relation to thoracolumbar myelopathies, canine and feline clinical patients with cervical myelopathies have been described with signs suggestive of SS as well. Although SS was not specifically mentioned, Forterre et al. (67) mentioned depressed thoracic limb withdrawal reflexes in dogs with cervical IVDE (67). The authors discussed pathophysiological mechanisms implied in SS as a postulated cause (67). Importantly, the subjectivity of the interpretation of the withdrawal reflex holds another possible explanation for a withdrawal reflex to be called “depressed” but the authors reported that they considered interobserver variation in technique (and interpretation implied) unlikely. In any case, Forterre et al. (67) have often been cited in relation to the unreliability of the clinical neurological examination, specifically the thoracic limb withdrawal reflexes, to differentiate between a C1–5 and C6–T2 myelopathy (67). Beltran et al. reported a dog with acute onset of decreased to absent withdrawal reflexes in all four limbs and decreased patellar reflexes in both pelvic limbs that was diagnosed with a C4–5 hydrated nucleus pulposus extrusion (HNPE) (61). Additionally, two dogs were reported to be localized to C6–T2 (presumably based on decreased/absent thoracic limb withdrawal reflexes) while they were diagnosed with a C3–4 HNPE in that same study (61). In another report, for a cat with an ischemic myelopathy at the level of C2, decreased withdrawal reflexes in the thoracic limbs led to a mislocalization to C6–T2 as well (68). These literature examples indicate that signs suggestive of SS are clinically recognized in cervical SCI. This phenomenon warrants future clinical scrutiny and study.

Moreover, the possibility of asymmetry of clinical findings in patients with SS is something that is not particularly mentioned or discussed in veterinary literature to the authors' knowledge. Nevertheless, it can be gleaned from descriptions in studies like that of Togawa et al. (60, 69) that it does occur (60, 69). In those studies, authors classified patients that exhibited either bi- or unilateral depressed withdrawal reflexes as patients that exhibited spinal shock. There is no particular emphasis on this possibility in other medical literature to the authors knowledge.

The clinical observation of the phenomenon of SS in patients with cervical SCI and possibility of asymmetry of SS can be important, as this illustrates that depressed spinal reflexes in thoracic and/or pelvic limbs may occur with cervical SCI and that even when spinal reflexes are depressed asymmetrically, an upper motor neuron lesion with SS should still be considered as a possible neuroanatomical localization to account for the signs in that patient.

### 3.2 Crossed extensor reflex

In short, the veterinary literature contains mostly anecdotal observations and descriptions concerning the crossed extensor reflex. While it is oftentimes but anecdotally associated with chronic spinal cord lesions (either due to a chronic disease process or chronic disruption of upper motor neuron pathways secondary to a prior acute SCI), there is extremely little actual clinical evidence in dogs in cats regarding its frequency, timing, and potential clinical value/importance. The presence of crossed extensor reflex in dogs that developed spinal walking after SCI is common (70). Although it is considered to have no prognostic value (20), in cats, it was significantly associated with a higher likelihood of regaining functional ambulatory status and the development of spinal walking (71).

Importantly, the crossed extensor reflex has been reported in acute spinal cord lesions, such as trauma, vascular events and intervertebral disc diseases (71–73). In these references, however, it is not always entirely clear in what time frame since the acute myelopathy the reflex was observed. Crossed extensor reflex was also reported in dogs affected by canine neuroangiostrongyliasis (Angiostrongylus cantonensis) (74) and in adult Nova Scotia Duck Tolling Retrievers diagnosed with a suspected hereditary degenerative encephalopathy (75). The former illustrates that the presence of this reflex should not be related to specific, common spinal cord pathology but that various spinal cord diseases can result in its presence. The latter illustrates that it might well be that involvement of upper motor neuron cell bodies themselves might account for a crossed extensor reflex when observed in patients with no overt spinal cord disease.

Of note, purposeful struggling of patients in lateral recumbency might be confused with crossed extensor reflex activity and should be taken into account when documenting the presence or absence of this reflex (20).

Clinically, crossed extensor reflex may be observed in the pelvic limbs with lesions in the thoracolumbar (T3–L3) or cervicothoracic (C6–T2) spinal cord segments, and in all limbs with lesions in the cranial cervical (C1–C5) spinal cord segments, but the presence of this reflex is not associated with lesions in the lumbosacral and caudal spinal cord segments (20). In fact, in the veterinary textbooks, the presence of crossed extensor reflex is often strictly associated with lesions cranial to the lumbar intumescence although there is a lack of evidence to support this statement (i.e. it may have been predominantly described for lesions affecting the upper motor neuron pathways, but pathology affecting the intumescences is at least theoretically possible) (18–20, 76, 77).

The clinical observation of the crossed extensor reflex in a patient with acute SCI or in a patient with a lesion affecting the lumbosacral intumescence or segments caudally to the intumescence can be important, as this illustrates that the presence of this reflex alone should not be necessarily relied upon to conclude on the chronicity and neuroanatomical localization of the lesion.

### 3.3 Mass reflex

The current clinical and academic understanding of mass reflexes largely relies on anecdotal evidence, limited textbook references, and extrapolation from human data. While several authors describe mass reflexes as expected findings in chronic upper motor neuron lesions, they provide little guidance regarding their timing, frequency, or diagnostic utility (78, 79). A prospective study identified reflex depression in 26% of dogs with acute thoracolumbar SCI but did not report mass reflexes (defined as: tail wag, urination, defecation, or stepping motions elicited by stimuli applied to the feet) at initial presentation while their study design would likely have enabled reporting thereof should they have observed this (58). This supports the notion that reflex depression (spinal shock) may be more typical in early SCI.

Veterinary clinicians and neurology specialists have anecdotally suggested that mass reflexes might imply chronicity and lesion severity, although no current evidence substantiates their use as prognostic indicators. Nociception (deep pain perception) remains the most reliable prognostic marker in canine SCI (with most studies focusing on intervertebral disc herniation as a cause of SCI) (80–82). Nevertheless, the perception that mass reflexes indicate chronic lesions can influence clinical decisions, particularly in cases in which an adequate history is not available. For instance, the presence of a mass reflex in a paraplegic, nociception-negative patient with an unknown timeline may lead clinicians to infer chronic injury and a graver prognosis (indirectly based on an assumption of chronically absent nociception), despite insufficient evidence supporting such assumptions.

The clinical observation of mass reflex activity in a patient with an acute SCI and mass reflex activity in conjunction with signs of SS can be of clinical importance and this illustrates that the mass reflex alone should not be necessarily relied upon to conclude on the chronicity of the lesion.

## 4 Clinical observations

### 4.1 Spinal shock

In this section, we present our observations in clinical patients that highlight the possibility of SS in naturally occurring cervical myelopathies as well as the possibility of asymmetry in its presentation. These aspects are relevant to consider for accurate neuroanatomical localization of spinal cord lesions in veterinary patients.

#### 4.1.1 Spinal shock with asymmetry in dogs with a C1–5 myelopathy

**Figure 1:**
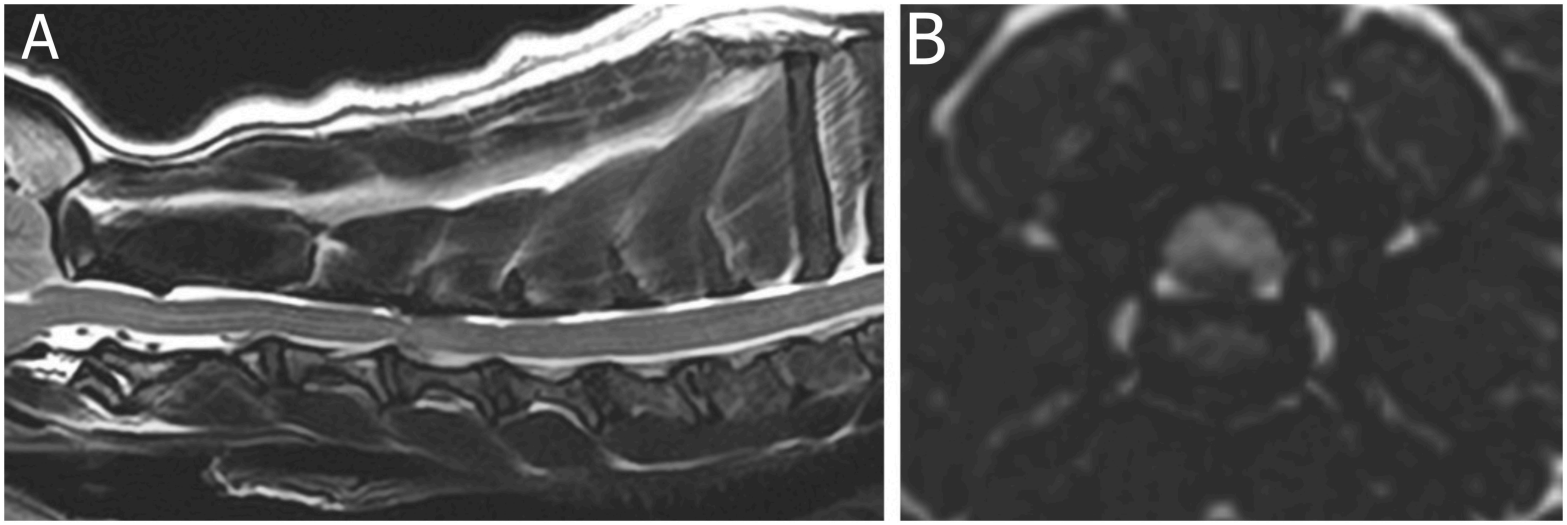
Magnetic resonance images (1.5T Canon Vantage Elan) of a French bulldog presented with a 24-h history of acute non-ambulatory tetraparesis, neck pain, dyspnea, and dysphonia. Muscle tone was reduced in the thoracic limbs and right pelvic limb. Absent to reduced withdrawal reflexes were noted in the thoracic limbs and right pelvic limb, attributed to spinal shock. **(A)** Sagittal T2W image of the cervical region. **(B)** Transverse 3DT2W reconstruction image at the level of the C3–4 intervertebral disc space. A right lateralized C3–C4 intervertebral disc extrusion (the material was predominantly ventrolateral to the spinal cord on the right side).

A 3-year-old French Bulldog presented with a 24-h history of acute non-ambulatory tetraparesis, neck pain, dyspnea, and dysphonia (Supplementary Video 1). Muscle tone was reduced in the thoracic limbs and right pelvic limb. Absent to reduced withdrawal reflexes were noted in the thoracic limbs and right pelvic limb. Cervical hyperesthesia was not obvious upon presentation. MRI (1.5T Canon Vantage Elan) of the cervical spinal cord showed a right lateralized C3–C4 intervertebral disc extrusion (the material was predominantly ventrolateral to the spinal cord on the right side) (Figure 1). The reduced muscle tone and spinal reflexes were attributed to SS. The dog was surgically treated via a standard ventral slot approach, and on a 14-day recheck, muscle tone and spinal reflexes were normal in all limbs.

**Figure 2:**
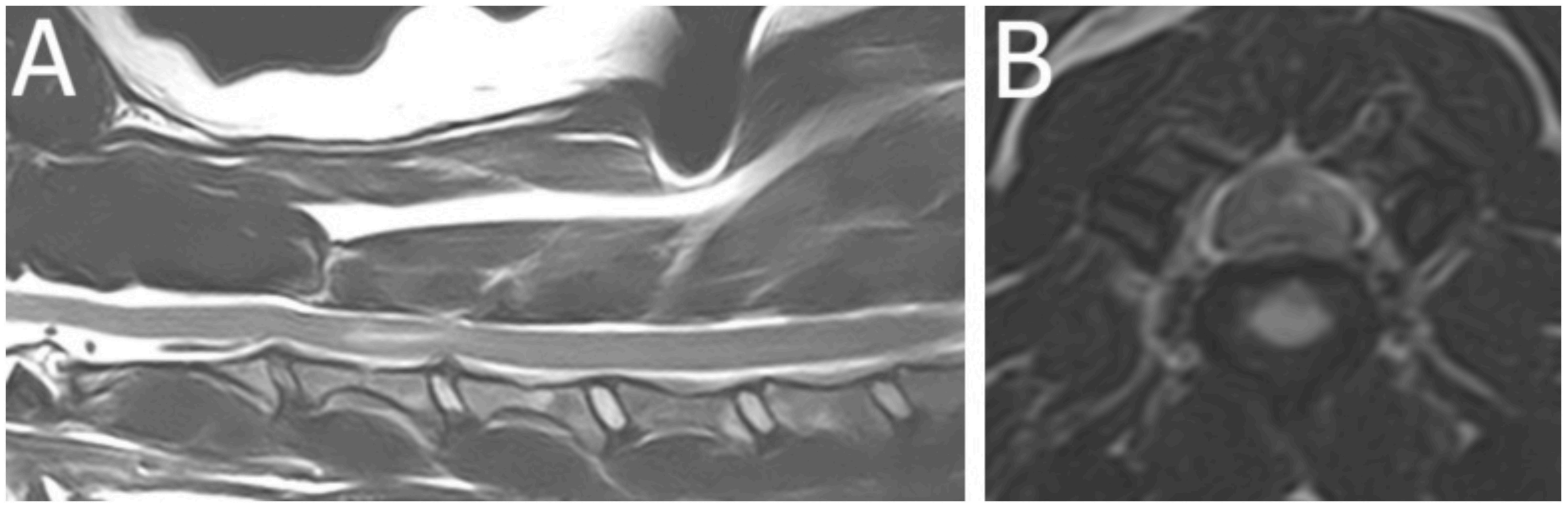
Magnetic resonance images (1.5T Canon Vantage Elan) of an 11-year-old Dutch Kooijkerdog was presented with a 36-h history of acute left-lateralized non-ambulatory tetraparesis. Muscle tone was absent in the left thoracic limb and increased in the left pelvic limb. Patellar hyperreflexia and decreased withdrawal reflex were present in the left pelvic limb. Partial Horner syndrome was present on the left side. **(A)** Sagittal T2W image of the cervical region. **(B)** Transverse T2W image at the level of the C3–4 intervertebral disc space. This dog was diagnosed with a partially HNPE C3–4 with left-lateralized intramedullary hyperintensity consistent with edema, contusion, or ischemic myelopathy.

An 11-year-old Dutch Kooijkerdog was presented with a 36-h history of acute left-lateralized non-ambulatory tetraparesis (Supplementary Video 2). Muscle tone was absent in the left thoracic limb and increased in the left pelvic limb. Patellar hyperreflexia and decreased withdrawal reflex were present in the left pelvic limb. Partial Horner syndrome was present on the left side. Based on MRI (1.5T Canon Vantage Elan), this dog was diagnosed with a partially HNPE C3–4 with left-lateralized intramedullary hyperintensity consistent with edema, contusion, or ischemic myelopathy (Figure 2). Follow-up 1 month later revealed almost complete recovery with persistent mild left-sided spastic paresis and proprioceptive deficits (thoracic limb more affected than the pelvic limb).

#### 4.1.2 Spinal shock with asymmetry in a dog with a T3–L3 myelopathy

**Figure 3:**
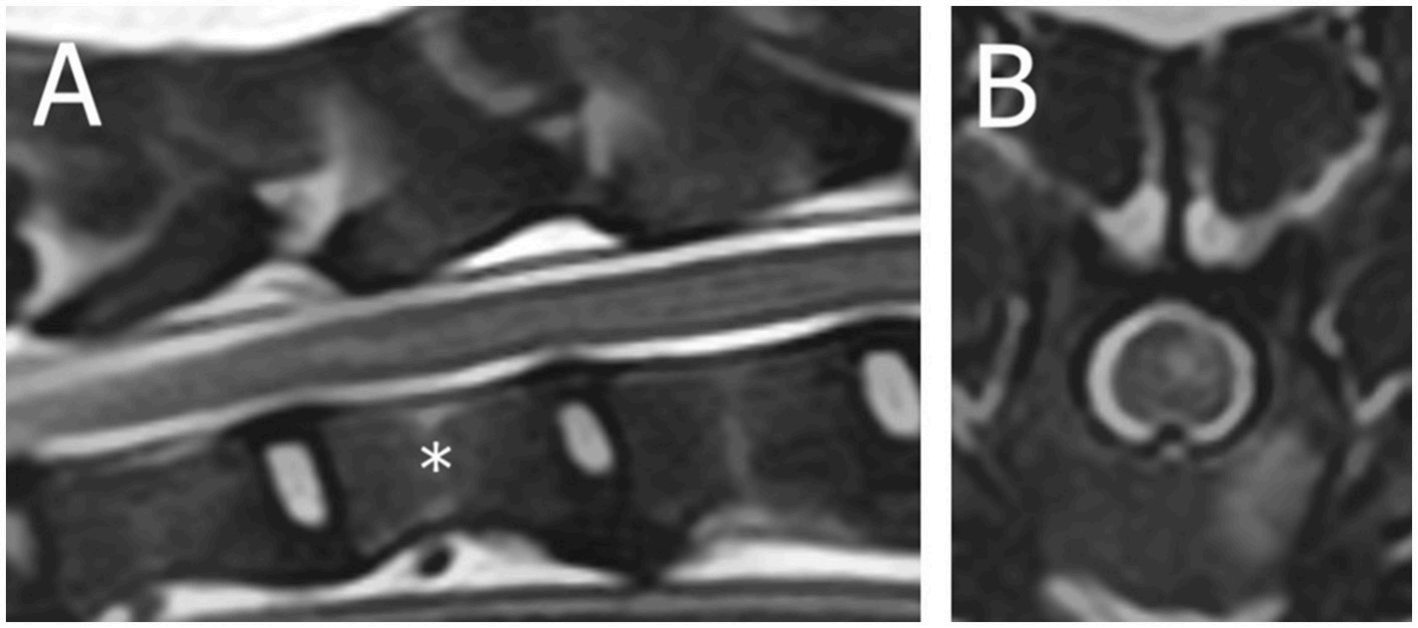
Magnetic resonance images (1.5T Canon Vantage Elan) of a 6-year-old female neutered crossbreed presented for acute non-ambulatory left-lateralized paraparesis with asymmetrical left-lateralized spinal shock (decreased withdrawal reflex of the left pelvic limb, retained patellar reflex) diagnosed with a left-sided caudal thoracic ischemic myelopathy. **(A)** Sagittal T2W image of the T11-T13 region (* = T12 vertebral body). **(B)** Transverse T2W image at the level of T12.

A 6-year-old female neutered crossbreed presented for acute non-ambulatory left-lateralized paraparesis with asymmetrical left-lateralized spinal shock (decreased withdrawal reflex of the left pelvic limb, retained patellar reflex) diagnosed [based on MRI (1.5T Canon Vantage Elan)] with a left-sided caudal thoracic ischemic myelopathy (Figure 3, Supplementary Video 3). Upon follow-up 2 weeks later via the referring veterinarian, the dog was ambulatory with mild paraparesis and ataxia.

### 4.2 Crossed extensor reflex

In this section, we present our observations in clinical patients that highlight the possibility of a crossed extensor reflex with a lesion at the lumbar intumescence, the possibility of a crossed extensor reflex in patients presenting in the acute phases after SCI, and the possibility of asymmetry in crossed extensor reflex activity. These aspects are relevant to consider for accurate inferences on chronicity and neuroanatomical localization of spinal cord lesions in veterinary patients.

#### 4.2.1 Crossed extensor reflex in a cat with a lesion of the lumbosacral intumescence

**Figure 4:**
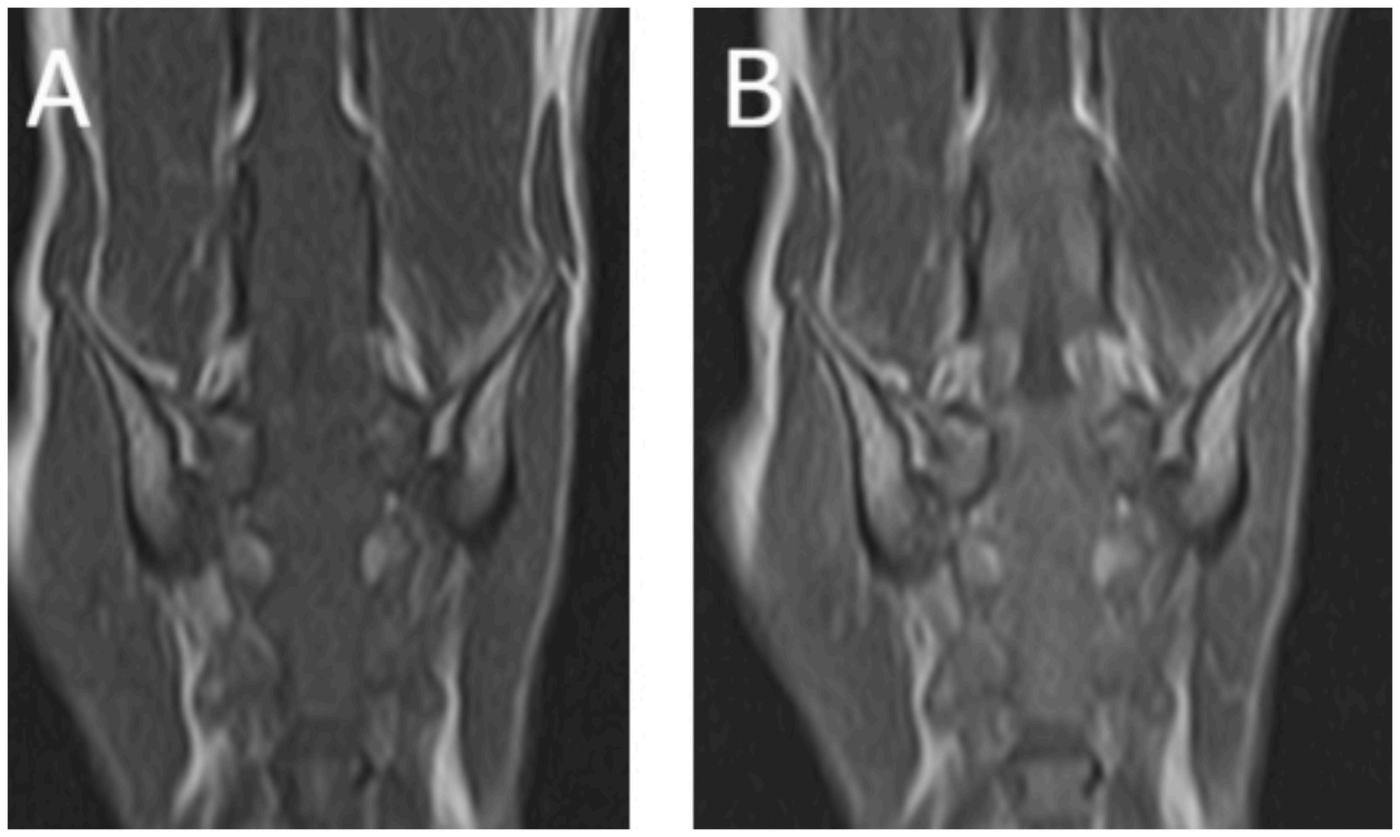
Magnetic resonance images (1.5T Canon Vantage Elan) of a 13-year-old male castrated European shorthaired cat showing crossed extensor reflex in the pelvic limbs due lymphoma affecting the L6-Cd spinal cord segments and nerves of the cauda equina. **(A)** Dorsal T1W pre-contrast at the level of the lumbosacral area. **(B)** Dorsal T1W post-contrast at the level of the lumbosacral area.

A 13-year-old male castrated European shorthaired cat was diagnosed with lymphoma affecting the L6-caudal spinal cord segments and cauda equina based on MRI (1.5T Canon Vantage Elan) and histopathological examination (Figure 4). The cat exhibited a crossed extensor reflex of the pelvic limbs (Supplementary Video 4).

#### 4.2.2 Crossed extensor reflex in a cat with an acute T3–L3 myelopathy

A 3-year-old mixed breed cat presented for acute-onset paraplegia. Based on radiographs, the cat was diagnosed with a traumatic thoracolumbar myelopathy (T13–L1 vertebral fracture/luxation). A crossed extensor reflex in both pelvic limbs was recorded 24-h after the traumatic event (Supplementary Video 5).

#### 4.2.3 Crossed extensor reflex in a dog with an acute cervical myelopathy

**Figure 5:**
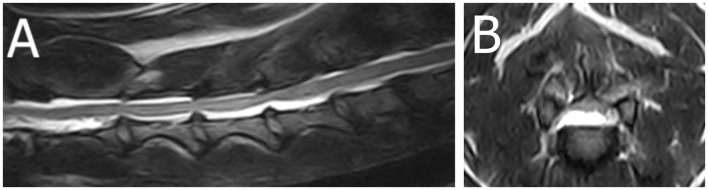
Magnetic resonance images (0.25T Esaote Vet-MR Grande) of a 6-year-old male castrated Weimaraner dog was diagnosed with a C4–5 HNPE. The clinical examination revealed non-ambulatory spastic tetraparesis with normal spinal reflexes and a crossed extensor reflex in both the pelvic and thoracic limbs. **(A)** Sagittal T2W image of the cervical region. **(B)** Transverse T2W image at the level of the C4–5 intervertebral disc space.

A 6-year-old male castrated Weimaraner dog presented for non-ambulatory spastic tetraparesis with normal spinal reflexes and a crossed extensor reflex in both the pelvic and thoracic limbs. (Supplementary Video 6). Based on MRI (0.25T Esaote Vet-MR Grande), the dog was diagnosed with a C4–5 HNPE (Figure 5).

#### 4.2.4 Asymmetrical crossed extensor reflexes of the thoracic limbs in a dog with an acute cervical myelopathy

**Figure 6:**
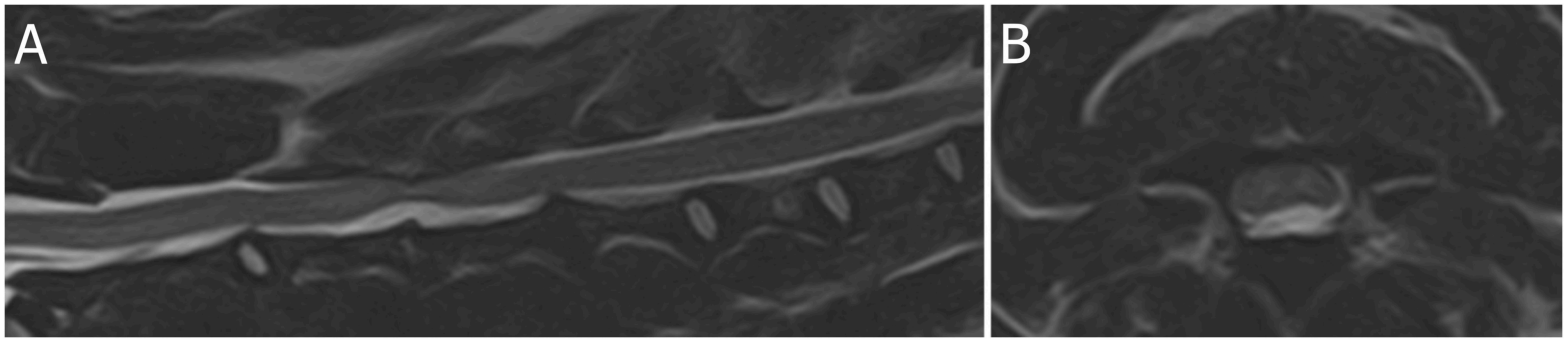
Magnetic resonance images (1.5T Canon Vantage Elan) of a 7-year-old female neutered crossbreed dog presented one day after acute-onset of spastic tetraplegia. The dog was diagnosed with a C3–4 HNPE (left worse than right), with a crossed extensor reflex of the thoracic limbs, most prominently of the left thoracic limb when the right thoracic limb withdrawal reflex is tested. **(A)** Sagittal T2W image of the cervical region. **(B)** Transverse T2W image at the level of the cranial endplate of C4.

A 7-year-old female neutered crossbreed dog presented 1 day after acute-onset of spastic tetraplegia (Supplementary Video 7). The dog was diagnosed with a C3–4 HNPE (left worse than right), with a crossed extensor reflex of the thoracic limbs, most prominently of the left thoracic limb when the right thoracic limb withdrawal reflex is tested (Figure 6).

### 4.3 Mass reflex

In this section, we present our observations in clinical patients that highlight the possibility that mass reflexes can occur in the acute phase of a thoracolumbar SCI and the possibility that these reflexes may be present simultaneously with spinal shock. These aspects are relevant to consider for accurate inferences on chronicity and neuroanatomical localization of spinal cord lesions in veterinary patients.

#### 4.3.1 Mass reflex in a dog with an acute T3–L3 myelopathy

**Figure 7:**
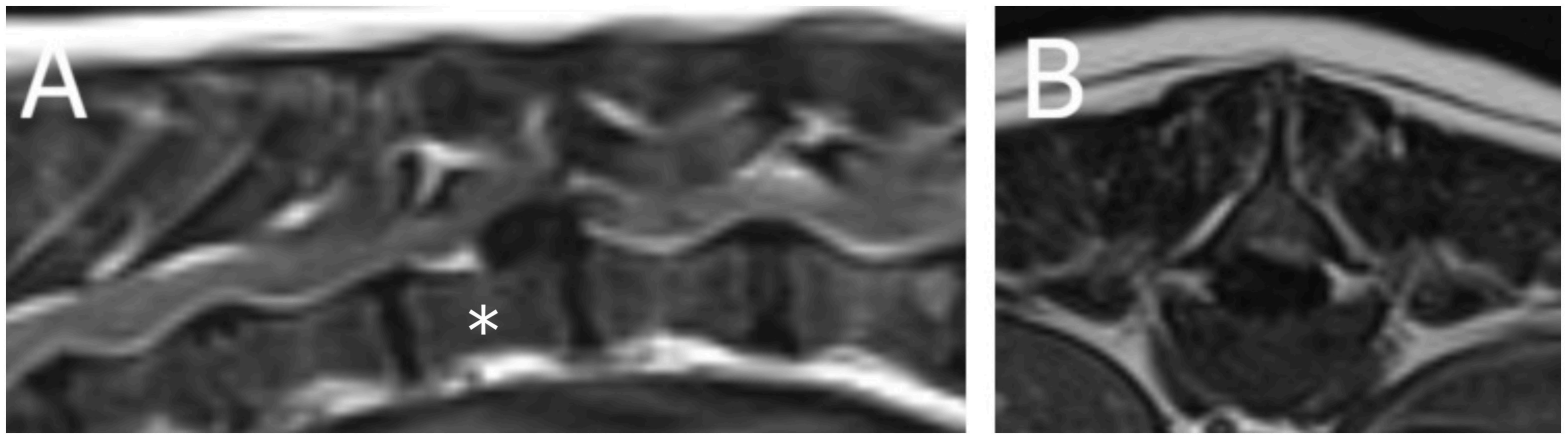
Magnetic resonance images (1.5T, Magnetom, Essenza, Siemens) of a 5-year-old, male neutered Dachshund diagnosed with a T11–T12 IVDE was presented with a 24-h history of ambulatory paraparesis and thoracolumbar hyperesthesia which progressed to paraplegia with absent nociception on the day of presentation. Neurological examination showed spastic paraplegia with absent nociception in both pelvic limbs, tail and perineal region. Patellar and withdrawal reflexes in pelvic limbs were intact bilaterally. There was a cutaneous trunci reflex cut-off at L1. Upon testing cutaneous trunci reflex, contralateral pelvic limb flexion and ipsilateral tail flexion was observed. **(A)** Sagittal T2W image of the T9-T13 region (* = T11 vertebral body). **(B)** Transverse T2W image at the level of the T11 endplate.

A 5-year-old, male neutered Dachshund diagnosed with a T11–T12 IVDE (based on MRI, Figure 7 (1.5T Magnetom Essenza, Siemens) was presented with a 24-h history of ambulatory paraparesis and thoracolumbar hyperesthesia which progressed to paraplegia with absent nociception on the day of presentation. Neurological examination showed spastic paraplegia with absent nociception in both pelvic limbs, tail and perineal region. Patellar and withdrawal reflexes in pelvic limbs were intact bilaterally. There was a cutaneous trunci reflex cut-off at L1. Upon testing cutaneous trunci reflex, contralateral pelvic limb flexion and ipsilateral tail flexion was observed (Supplementary Video 8).

#### 4.3.2 Mass reflexes concurrently with spinal shock in two dogs with acute T3–L3 myelopathies

**Figure 8:**
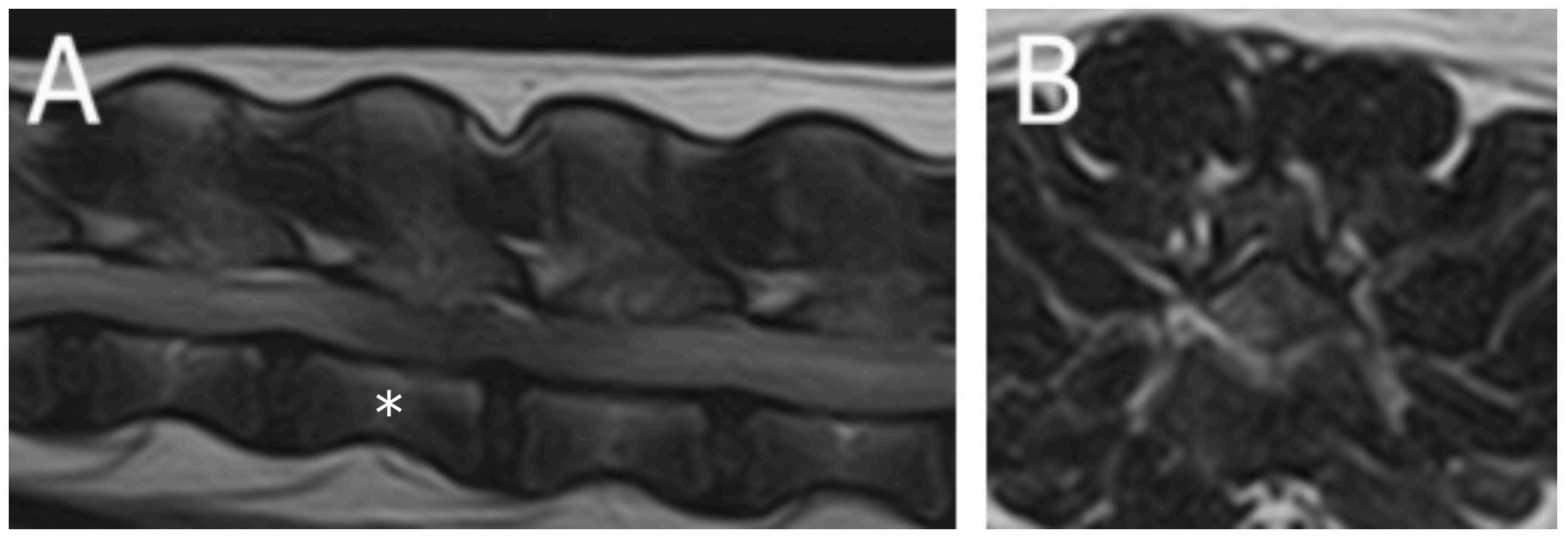
Magnetic resonance images (1.5T Canon Vantage Elan) of a 6-year-old male neutered crossbreed dog was diagnosed with an L2–L3 IVDE. It was presented flaccid pelvic limb muscle tone, absent patellar, sciatic and withdrawal reflexes, an areflexic perineal region, a flaccid tail, and Schiff-Sherrington posture. Nociception was absent in the pelvic limbs and tail (i.e. the dog was “deep pain negative”). Upon stimulation with hemostatic forceps, the tail exhibited rhythmic wagging and tonic flexion, consistent with mild mass reflex activity. **(A)** Sagittal T2W image of the L1–L4 region (* = L2 vertebral body). **(B)** Transverse T2W image at the level of the L1–2 intervertebral disc space.

A 6-year-old male neutered crossbreed dog was diagnosed with an L2-L3 IVDE based on MRI (1.5T Canon Vantage Elan) (Figure 8, Supplementary video 9). It was presented flaccid pelvic limb muscle tone, absent patellar, sciatic and withdrawal reflexes, an absent perineal reflex on the left and markedly reduced perineal reflex on the right, a flaccid tail, and Schiff-Sherrington posture. Nociception was absent in the pelvic limbs and tail (i.e. the dog was “deep pain negative”). Upon stimulation of the tail with hemostatic forceps, the tail exhibited rhythmic wagging and tonic flexion, consistent with mass reflex activity.

**Figure 9:**
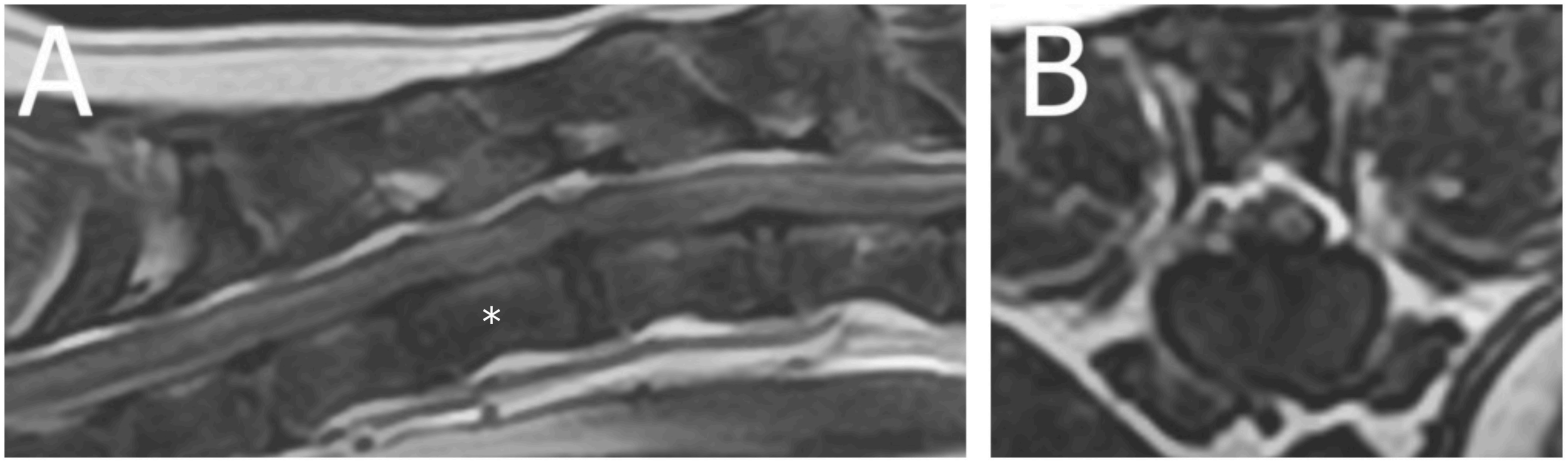
Magnetic resonance images (1.5T Canon Vantage Elan) of a 7-year-old male neutered crossbreed dog was diagnosed with a T12–T13 IVDE with concurrent epidural hemorrhage. The dog showed signs consistent with spinal shock (reduced to absent spinal reflexes in the pelvic limbs – not depicted in the video). Pelvic limb muscle tone was reduced. Stimulation with forceps of the pelvic limb digits elicited delayed but briefly sustained pelvic limb (hip) and tail flexion, indicative of a mass reflex despite the presence of spinal shock. **(A)** Sagittal T2W image of the thoracolumbar region (* = T12 vertebral body). **(B)** Transverse T2W image at the level of the T12–13 intervertebral disc space.

A 7-year-old male neutered crossbreed dog was diagnosed with a T12–T13 IVDE with concurrent epidural hemorrhage based on MRI (1.5T Canon Vantage Elan) (Figure 9, Supplementary Video 10). The dog showed signs consistent with spinal shock (reduced to absent spinal reflexes in the pelvic limbs—not depicted in the video). Pelvic limb muscle tone was reduced. Stimulation with forceps of the pelvic limb digits elicited delayed but briefly sustained pelvic limb (hip) and tail flexion, indicative of a mass reflex despite the presence of spinal shock.

## 5 Discussion

This review analyzes and discusses current literature regarding well-known neuro(patho)physiological phenomena, traditionally thought to pertain to (per)acute myelopathies, such as SS, or chronic myelopathies, such as crossed extensor reflex and mass reflex. We also reported clinical observations that somehow challenge existing theories in scientific literature or highlight underreported conditions, such as asymmetrical SS, asymmetrical crossed extensor reflex, crossed extensor and mass reflexes in patients presenting with peracute myelopathies, mass reflexes concurrent with signs of SS, and pelvic limb crossed extensor reflex in patients with pathology affecting the lumbosacral intumescence.

With regard to asymmetrical SS, Hodshon and Thomas mention the possibility of a unilateral condition in 2/9 cases that had depressed withdrawal reflexes (58). Other sources in literature sporadically do contain descriptions of signs that can be interpreted as signs of unilateral or asymmetrical SS, although not specifically referred to as such (60, 69). Speculatively, etiologies of SCI like ischemic myelopathy could more likely account for unilateral SS than IVDE. However, this cannot yet be supported based on currently available literature. SS (especially when asymmetrical) is possibly underdiagnosed, misdiagnosed or underreported due to its variable presentation, perceived clinical irrelevance, and/or mimicry of lower motor neuron disease. Understanding this phenomenon is important for accurate neurological localization, formulating a differential diagnosis list, and correctly performing diagnostic imaging on the region of interest.

This review also discussed and presented observations of crossed extensor reflexes and mass reflexes in clinical veterinary neurology. These two reflexes are traditionally associated with chronicity of lesions (and sometimes severity of lesions) but occasionally associated with acute spinal cord lesions in veterinary literature—although such references do not always specifically state the time frame within which these reflexes developed—, challenging the classical clinical picture as do our clinical observations as described (71, 83).

In dogs and cats, the first phase of SS may be very brief (i.e. minutes to hours) (12, 58, 65). Thus, while true hyperreflexia (spasticity) traditionally requires days to develop, dogs may exhibit reflex return within hours, that can be interpreted as early improvement of SS. This early return of reflex activity may also influence early occurrence of hyperreflexia such as crossed extensor reflex and mass reflex activity. In our cases that displayed early onset of crossed extensor reflexes, we cannot exclude pre-existing chronic upper motor neuron lesions, with spinal circuits already disinhibited. But this seems less likely and not supported by imaging findings in those cases. In a recent review, it is discussed that preexisting subclinical myelopathy (in elderly people) can contribute to the rapid recovery of reflexes (although mainly referring to deep tendon reflexes) (2). Also, polysynaptic cutaneous reflexes that receive less supraspinal facilitation and/or synaptic areas are typically less affected by SS (2). The authors also suggest that the evolution of reflexes over several days following injury may be more relevant to prognosis than the presence or absence of reflexes on the day of injury (2). This consideration may be true for veterinary SCI patients as well. Indeed, the clinical observations that we present in this review could, when considered in isolation, potentially lead to unfounded assumptions regarding prognosis. The observation of the development of the reflex activity over time may yield different interpretations. Caution is therefore advised in the acute phase of SCI when interpreting spinal reflex activity regarding prognosis.

The presence of mass reflex concurrent with signs of SS that we observed in clinical patients highlights an unusual clinical picture clinicians should be aware of. The reported observations overall may suggest that the presence of a crossed extensor and mass reflex in a patient should not be interpreted as indicating chronicity or poor prognosis in isolation, but it must be evaluated in the context of other reflexes, muscle tone, nociception, and imaging findings.

Lastly, we presented a patient displaying pelvic limb crossed extensor reflex with a lesion localized in the lumbar intumescence. This particular case challenges the traditional paradigm by showing crossed extensor reflex with lesion localization different from what was expected, i.e. a T3–L3 myelopathy might be expected in such a patient. Merlet et al. (83) suggest that SCI can lead to a loss of functional specificity, increased reflex gain, and reorganization of local spinal networks. Hypothetically, somatosensory feedback from the lumbar and perineal regions, particularly when altered by chronic disease in or caudal to the lumbosacral intumescence, could therefore potentially modulate spinal reflex circuits resulting in a crossed extensor reflex. Additionally, bidirectional interactions between lumbar and sacral central pattern generators and overlapping afferent projections may contribute to abnormal interlimb reflex activity (83). We hypothesize that local circuit disinhibition and maladaptive plasticity likely explain the presence of this reflex despite the atypical lesion localization in this feline patient. Further insights from experimental studies support the possibility of crossed extensor reflex expression in atypical spinal cord locations by highlighting pathomechanistic details and anatomical considerations (27, 84). Matthes and Ruch demonstrated that even a single, weak stimulus could elicit contralateral extensor muscle activity in chronic spinal cats, with reflex latency varying widely from 8 to 60 ms (27). This variability suggests that post-injury changes in spinal excitability and interneuronal processing, not rigid anatomical localization, may govern reflex expression. More recently, LaFlamme et al. (84) showed that, in mice, crossed reflexes can be mediated by distributed excitatory interneuronal networks, rather than solely by classical commissural pathways. Hypothetically, these networks, when functionally altered by injury, could enable the emergence of interlimb reflexes in the absence of true upper motor neuron lesions. Physiological studies show that, over time, neuroplastic changes can occur in the spinal cord below the lesion, some reflex arcs can reorganize and local spinal circuits can become hyperexcitable; experimental work shows partial preservation or recovery of crossed extensor reflexes following peripheral and central nervous system injuries in animals, often via sprouting, regeneration, and reorganization of afferent fibers and interneuronal circuits (85–88). Focal, incomplete lesions of the lumbosacral intumescence itself may in part also lead to similar processes. To explain the unusual clinical finding in our feline patient, we can speculate that a partial lower motor neuron lesion may have left enough interneuronal circuits intact to enhance crossed extensor reflex activity or a concurrent upper motor neuron lesion was present in our patient. As already mentioned, somatosensory feedback from the lumbar and perineal regions can modulate segmental spinal reflex circuits and can amplify reflexes (83), possibly also when lesions affect those segments themselves as in the patient that we described here.

Overall, our observations and the existing body of literature, veterinary, experimental, and human medical literature, serve to advise caution in the interpretation of spinal reflexes; they should be evaluated and interpreted in the context of other clinical findings and exceptions to the ‘rule' are possible.

## 6 Conclusion

In conclusion, this review discussed the phenomena of spinal shock, crossed extensor reflex, and mass reflexes in dogs and cats. The authors presented uncommonly reported clinical observations with regard to these phenomena in veterinary patients. The interpretation of spinal reflexes and these phenomena may impact clinical evaluation, neuroanatomical localization, differential diagnosis list compilation and diagnostic work-up, and lead to inferences of severity and chronicity of the myelopathies, and prognostication. As there is yet much left to study regarding these phenomena and their value for all of these—if any—caution is advised to draw firm conclusions based on these observations in isolation. Future studies evaluating and reporting these observations more systematically are encouraged.

## Supplementary material

Supplementary Video 1 A 3-year-old French bulldog presented with a 24-h history of acute non-ambulatory tetraparesis, neck pain, dyspnea, and dysphonia. Muscle tone was reduced in the thoracic limbs and right pelvic limb. Absent to reduced withdrawal reflexes were noted in the thoracic limbs and right pelvic limb, attributed to spinal shock. The dog was diagnosed with a right-lateralized C3–C4 intervertebral disc extrusion.

Supplementary Video 2 An 11-year-old Dutch Kooijkerdog was presented with a 36-h history of acute left-lateralized non-ambulatory tetraparesis. Muscle tone was absent in the left thoracic limb and increased in the left pelvic limb. Patellar hyperreflexia and decreased withdrawal reflex were present in the left pelvic limb. Partial Horner syndrome was present on the left side. This dog was diagnosed with a partially HNPE C3–4 with left-lateralized intramedullary hyperintensity consistent with edema, contusion, or ischemic myelopathy.

Supplementary Video 3 A 6-year-old female neutered crossbreed presented for acute non-ambulatory left-lateralized paraparesis with asymmetrical left-lateralized spinal shock (decreased withdrawal reflex of the left pelvic limb, retained patellar reflex) diagnosed with a left-sided caudal thoracic ischemic myelopathy.

Supplementary Video 4 A 13-year-old male castrated European shorthaired cat showing crossed extensor reflex in the pelvic limbs due lymphoma affecting the L6-Cd spinal cord segments and nerves of the cauda equina.

Supplementary Video 5 A 3-year-old mixed breed cat presented for acute-onset paraplegia. The cat was diagnosed with a traumatic thoracolumbar myelopathy (T13–L1 vertebral fracture/luxation). A crossed extensor reflex in both pelvic limbs was recorded 24-h after the traumatic event.

Supplementary Video 6 A 6-year-old male castrated Weimaraner dog was diagnosed with a C4–5 HNPE. The clinical examination revealed non-ambulatory spastic tetraparesis with normal spinal reflexes and a crossed extensor reflex in both the pelvic and thoracic limbs.

Supplementary Video 7 A 7-year-old female neutered crossbreed dog presented 1 day after acute-onset of spastic tetraplegia. The dog was diagnosed with a C3–4 HNPE (left worse than right), with a crossed extensor reflex of the thoracic limbs, most prominently of the left thoracic limb when the right thoracic limb withdrawal reflex is tested.

Supplementary Video 8 A 5-year-old, male neutered Dachshund diagnosed with a T11–T12 IVDE was presented with a 24-h history of ambulatory paraparesis and thoracolumbar hyperesthesia which progressed to paraplegia with absent nociception on the day of presentation. Neurological examination showed spastic paraplegia with absent nociception in both pelvic limbs, tail and perineal region. Patellar and withdrawal reflexes in pelvic limbs were intact bilaterally. There was a cutaneous trunci reflex cut-off at L1. Upon testing cutaneous trunci reflex, contralateral pelvic limb flexion and ipsilateral tail flexion was observed.

Supplementary Video 9 A 6-year-old male neutered crossbreed dog was diagnosed with an L2–L3 IVDE. It was presented flaccid pelvic limb muscle tone, absent patellar, sciatic and withdrawal reflexes, an areflexic perineal region, a flaccid tail, and Schiff-Sherrington posture. Nociception was absent in the pelvic limbs and tail (i.e. the dog was “deep pain negative”). Upon stimulation with hemostatic forceps, the tail exhibited rhythmic wagging and tonic flexion, consistent with mild mass reflex activity.

Supplementary Video 10 A 7-year-old male neutered crossbreed dog was diagnosed with a T12–T13 IVDE with concurrent epidural hemorrhage. The dog showed signs consistent with spinal shock (reduced to absent spinal reflexes in the pelvic limbs—not depicted in the video). Pelvic limb muscle tone was reduced. Stimulation with forceps of the pelvic limb digits elicited delayed but briefly sustained pelvic limb (hip) and tail flexion, indicative of a mass reflex despite the presence of spinal shock.
